# Cost implications of PSA screening differ by age

**DOI:** 10.1186/s12894-018-0344-5

**Published:** 2018-05-09

**Authors:** Karthik Rao, Stella Liang, Michael Cardamone, Corinne E. Joshu, Kyle Marmen, Nrupen Bhavsar, William G. Nelson, H. Ballentine Carter, Michael C. Albert, Elizabeth A. Platz, Craig E. Pollack

**Affiliations:** 1 Johns Hopkins University School of Medicine, Baltimore, MD USA; 2 Financial Analysis Unit, Johns Hopkins Health System, Baltimore, MD USA; 3 Department of Epidemiology, Johns Hopkins Bloomberg School of Public Health, Baltimore, MD USA; 4 Sidney Kimmel Comprehensive Cancer Center at Johns Hopkins, Baltimore, MD USA; 5 Johns Hopkins Health Care, Glen Burnie, Baltimore, MD USA; 6 Department of Urology and the James Buchanan Brady Urological Institute, Johns Hopkins University School of Medicine, Baltimore, MD USA; 7 Department of Environmental Health Sciences, Johns Hopkins Bloomberg School of Public Health, Baltimore, MD USA; 8 Johns Hopkins Community Physicians, Johns Hopkins Medical Institutions, Baltimore, MD USA; 9 Department of Medicine, Johns Hopkins University School of Medicine, 2024 E. Monument Street, Suite 2-519, Baltimore, MD 21287 USA

**Keywords:** Prostate cancer, Screening, Costs

## Abstract

**Background:**

Multiple guidelines seek to alter rates of prostate-specific antigen (PSA)-based prostate cancer screening. The costs borne by payers associated with PSA-based screening for men of different age groups—including the costs of screening and subsequent diagnosis, treatment, and adverse events—remain uncertain. We sought to develop a model of PSA costs that could be used by payers and health care systems to inform cost considerations under a range of different scenarios.

**Methods:**

We determined the prevalence of PSA screening among men aged 50 and higher using 2013-2014 data from a large, multispecialty group, obtained reimbursed costs associated with screening, diagnosis, and treatment from a commercial health plan, and identified transition probabilities for biopsy, diagnosis, treatment, and complications from the literature to generate a cost model. We estimated annual total costs for groups of men ages 50-54, 55-69, and 70+ years, and varied annual prostate cancer screening prevalence in each group from 5 to 50% and tested hypothetical examples of different test characteristics (e.g., true/false positive rate).

**Results:**

Under the baseline screening patterns, costs of the PSA screening represented 10.1% of the total costs; costs of biopsies and associated complications were 23.3% of total costs; and, although only 0.3% of all screen eligible patients were treated, they accounted for 66.7% of total costs. For each 5-percentage point decrease in PSA screening among men aged 70 and older for a single calendar year, total costs associated with prostate cancer screening decreased by 13.8%. For each 5-percentage point decrease in PSA screening among men 50-54 and 55-69 years old, costs were 2.3% and 7.3% lower respectively.

**Conclusions:**

With constrained financial resources and with national pressure to decrease use of clinically unnecessary PSA-based prostate cancer screening, there is an opportunity for cost savings, especially by focusing on the downstream costs disproportionately associated with screening men 70 and older.

**Electronic supplementary material:**

The online version of this article (10.1186/s12894-018-0344-5) contains supplementary material, which is available to authorized users.

## Background

Increasingly, health care systems are adopting risk-based payment strategies in which they are responsible for the health care costs incurred by their patients [1]. In this setting, health care systems are under pressure to provide evidence-based and efficient care for their beneficiaries. One area in which health systems may better align practice with evidence-based guidelines is cancer screening. Research suggests that many patients, including elderly patients and those with limited life expectancies, routinely receive cancer screening when they are unlikely to benefit [2]. The downstream costs of screening and subsequent treatment are substantial [3].

Recent guidelines for prostate specific antigen (PSA)-based prostate cancer screening have called for a reduction in the number of men who receive screening. The United States Preventive Services Task Force (USPTF) recommends against routine screening in all men (though draft guidelines may modify this) whereas the American Urological Association (AUA) advises against screening in men aged 70 or higher, except in those in the best health after shared decision making [4–6]. Early evidence suggests that the rate of PSA screening has decreased with a corresponding decline in prostate cancer incidence [7–9]. Nonetheless, many men aged 70 and higher continue to receive screening [10, 11]. While health systems may have an incentive to reduce clinically unnecessary PSA screening among their beneficiaries, the financial costs—from a health care system perspective—have not, to our knowledge, been quantified.

In this study, we tested how varying the rates of PSA screening among men of different age groups may influence associated health care expenditures. To do so, we generated a model using data from a large network of primary care providers in Maryland and Washington D.C. along with insurance claim data and estimates from the medical literature. These data were used to calculate the costs borne by payers associated with screening, subsequent diagnosis, treatment, and adverse events of prostate cancer. We then used our model to test how a number of different scenarios may impact costs including changing the prevalence of PSA screening among men in different age groups, increasing the proportion of men with low risk disease who undergo active surveillance, and changing the positive and negative predictive value of the screening test itself. By altering the rates of PSA screening or unit costs, our model is adaptable to different healthcare systems and with institution specific data, could be used to make cost conscientious decisions for system, provider, and patient interventions that seek to alter current patterns of PSA screening.

## Methods

We conducted a retrospective study to create a model of prostate cancer screening costs using data on (1) the prevalence of prostate cancer screening; (2) the reimbursed costs using procedure and complication reimbursement claims; and (3) the probabilities of screened patients developing and being treated for prostate cancer, and experiencing adverse events.

### Prostate cancer screening

We determined the annual prevalence of PSA screening among men aged 50 and higher who received primary care from a large, multispecialty group of ambulatory practices from April 1, 2013 to March 31, 2014 using electronic medical record data. Men with a known history of prostate cancer based on Internal Classification of Diseases (ICD)-9 code were excluded. The multispecialty group encompasses urban, suburban, and rural settings from an academic healthcare network in Maryland and Washington DC with over 39 clinic locations and 240,000 patients seen in the past year.

### Identification of procedure and complication costs

We estimated costs associated with screening and subsequent diagnosis, treatment, and complications using reimbursement claims data from a large, self-insured health plan in the mid-Atlantic region. Events of PSA screening, prostate biopsy, and treatment (radical prostatectomy and radiation) were identified by Current Procedural Terminology (CPT) codes (Additional file 1) in the claims database. The cost associated with each claim was the payment from the insurance carrier to the healthcare provider and does not include patients’ out-of-pocket, co-pay and secondary insurance payments. Data from 2004-2013 was employed to ensure enough patients for stable cost estimates; the time period ended in 2013 based on data availability at the time of analysis.

**Table 1:** Patient Stratification by Prostate Cancer Risk and Treatment Modality with Treatment and Complication Costs for Radical Prostatectomy and Radiotherapy

	Active Surveillance	Radical Prostatectomy	Radiotherapy	Cryotherapy	Primary Androgen Deprivation Therapy
%					
Low	9.2%	56.8%	23.3%	3.1%	7.6%
Intermediate	4.8%	52.9%	25.8%	4.5%	11.9%
High	3.2%	32.2%	25.6%	6.1%	32.8%
Unknown	9.9%	42.2%	26.3%	2.6%	18.9%
N					
Low	5	28	11	2	4
Intermediate	2	26	13	2	6
High	1	6	5	1	7
Unknown	2	7	4	0	3
Total	10	67	33	5	20
Total cost by treatment as a percentage of total cost of screening and treatment		31.3%	32.0%		
Complication rate		17.0%	1.6%		
Total complication cost by treatment as a percentage of total cost of screening and treatment		10.4%	0.11%		
Total treatment cost as a percentage of total screening and treatment cost	66.6%				

Source of cost estimate is insurance claim data from a commercial health plan in the mid-Atlantic

Active surveillance, cryotherapy, and primary androgen deprivation therapy are not included in total cost amount as they are not primary modalities of active therapy, which we focused our analysis on

We estimated the costs and rate of short-term complications resulting from prostate biopsy, radical prostatectomy, and prostate-targeted radiation therapy by identifying patients who utilized healthcare resources within 30 days (biopsy, radiation) or 90 days (prostatectomy) of the procedure and had ICD-9 diagnosis codes consistent with a complication for the procedure of interest (Additional file 2). The rates of complication were calculated by dividing the number of patients with a complication by the number of patients who had the procedure. To make our costs of complications less sensitive to outliers, we removed 5 patients whose costs were greater than 2 standard deviations from the mean. After outliers were removed, the average cost of treatment for complications from prostate biopsy, and prostate targeted radiation therapies from 2004-2013 was determined and inflated to 2013 dollars.

### Transition probabilities from prostate cancer screening to diagnosis and treatment

**Fig. 1:**
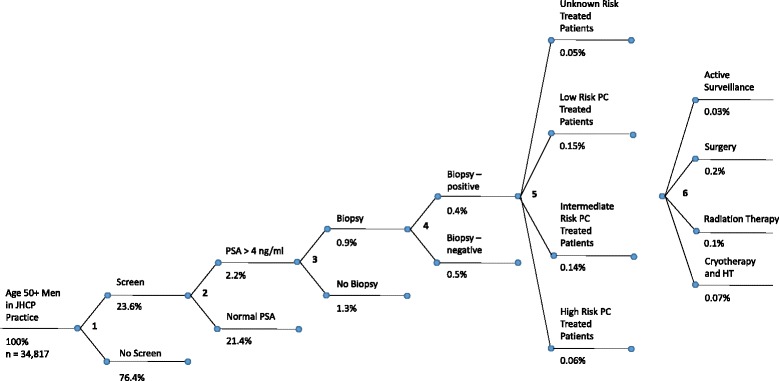
Node diagram of prostate cancer screening, diagnosis, and treatment pathway indicating the percentage of screen eligible patients who move through each node. **a** PSA screening practice data from a large, multispecialty group from April 1, 2013 to March 31, 2014 was used to model screening prevalence for age groups 50-54, 55-69, and 70+ years. **b** D’Amico low risk: PSA less than or equal to 10, a Gleason score less than or equal to 6, or are in clinical stage T1-2a. **c**. D’Amico intermediate risk: PSA between 10 and 20, a Gleason score of 7, or are in clinical stage T2b. **d**. D’Amico high risk: PSA more than 20, a Gleason score equal or larger than 8, or are in clinical stage T2c-3a. Note: Prostate Specific Antigen (PSA), Prostate Cancer (PC), Hormone Therapy (HT)

A node diagram (Fig. 1) was created to model a patient’s experience beginning with prostate cancer screening through treatment. For node 1, PSA screening practice data from patients treated by the multispecialty group on April 1, 2013 to March 31, 2014 was used to model screening prevalence. At each node, literature probabilities were used to determine the proportion of patients, by age group, who moved forward to the next node. The specific nodes that were modeled included the proportion of men who had a PSA greater than 4 ng/dl (node 2) [12], proportion of men who had a biopsy (node 3) [13], proportion of men who had a positive biopsy (node 4) [13], proportion of men classified by prostate cancer risk (node 5) [14], and proportion of men classified by treatment received (node 6) [14].

### Statistical analysis

For men who underwent screening during the 12-month period, we calculated the number of men who would be expected to progress through each node of the diagram. For each node, the per patient costs were multiplied by the total number of patients to determine the final costs of prostate cancer screening and treatment in the current scenario. We report the costs of the different lines of service/nodes as percentages of the total costs.

To understand the cost impact of changes in the prevalence of PSA-based prostate cancer screening, we developed scenarios in which we changed the screening prevalence during the 12 months among men of different age groups. In particular, we assessed PSA screening prevalences ranging between 5-50% in each age group (50-54, 55-69, 70+ years). We next sought to evaluate the impact of changing current patterns of treatment. With studies suggesting that older men with low risk disease frequently do well without active treatment [15], we modeled the cost implications of having more men 70+ with low risk disease (defined as PSA less than or equal to 10, a Gleason score less than or equal to 6, or are in clinical stage T1-2a by D’Amico criteria [16] and as modeled in node 5) move from radical prostatectomy to active surveillance.

Finally, new tools for prostate cancer screening are on the horizon which have the potential to (a) reduce the number of men without prostate cancer who undergo prostate biopsies (e.g., reduce the false positive rate of the screening test) and (b) increase the number of men with prostate cancer who screen positive (e.g., increase the true positive rate of the screening test). We modeled the potential cost implications of each scenario using a 10% reduction in the false positive rate and a 10% increase in the true positive rate as estimates of what a novel test may achieve. To estimate the total number of patients with prostate cancer for this analysis, we used literature estimates to determine the percentage of PSA screen positive patients with prostate cancer that may be missed by biopsy and also the percentage of patients with a negative PSA screen that may have prostate cancer. We assume that first biopsy misses 25% of positive prostate cancers [17] and that 15.2% of men with a negative PSA screen have prostate cancer [18].

## Results

We identified 34,817 men treated by the multispecialty group who were eligible for PSA-based prostate cancer screening; approximately 18% were ages 50-54, 52% were ages 55-69, and 30% were ages 70 and older. Of these, 8,213 (23.6%) were screened. The prevalence of screening varied by age group with men between the ages of 55-69 being the most likely to be screened (28.5%). In comparison, 17.8% of men ages 50-54 and 18.4% ages 70 and older were screened.

Based on current patterns of screening, we estimated that the costs of the PSA screening represented 10.1% of the total costs of screening, diagnosis, treatment, and complications for eligible patients. The costs of biopsies and associated complications were 23.3% of the total costs whereas the cost of treatment and associated complications were 66.6% of the total costs (Table 1). Although only 0.3% of all screen eligible patients were treated, they accounted for two thirds of all costs in the screening, diagnosis, treatment, and associated complications pathway. Complications from radical prostatectomy accounted for 10.4% of the treatment costs, whereas radiotherapy complications accounted for 0.1% and biopsy complications accounted for 5.4% of treatment costs. In the Additional file 3, we present the model we generated for the above analysis, which other health systems can use to estimate and partition costs. The following parameters can be modified: population size, screening rate among men of different age groups, and costs of treatment and associated complications.

**Fig. 2:**
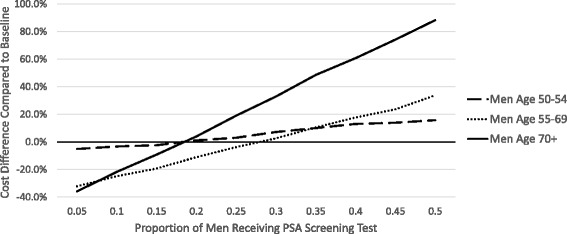
Change in the percentage of total costs of prostate cancer screening, diagnosis, treatment, and associated complications as a function of PSA screening prevalence among eligible men. Observed screening prevalence at the multispecialty group is represented by the point each line intersects with the x-axis

In Fig. 2, we show how different screening prevalences among men from different age groups influence costs. Increasing the prevalence of screening from the observed baseline in the multispecialty group (where the baseline prevalence is represented by the point each line intersects with the x-axis) is associated with increased costs from the current scenario. As the groups increased in age, there was a greater effect on overall cost from varying screening rate with the highest costs associated with changing screening amongst the oldest men. For each 5-percentage point increase in PSA screening among men 70 years and older, the total costs to the health care system increased by 13.8%. A 5-percentage point increase in PSA screening among men age 50-54 and 55-69 resulted in a smaller increase in total costs: 2.3% and 7.3% increase in total costs from the current scenario, respectively.

In assessing the cost impact of men age 70 and older with low risk prostate cancer choosing active surveillance instead of radical prostatectomy, we used $3,782 [19] as the cost of active surveillance over a 1-year time frame in contrast to the 30 day-time frame (radiation) and 90-day time frame (prostatectomy) used to determine the costs of active treatment. In our initial scenario, only 7.7% of the 26 men over 70 diagnosed with low risk disease were estimated to receive active surveillance. For every 10-percentage point increase in the proportion of low risk men 70+ who elect active surveillance, there would be a resulting 2.1% reduction in costs treating men in this age group and a more modest 1.1% reduction in total costs compared to the current scenario.

To determine the cost implications of a reduction in the false positive rate for a new theoretical prostate cancer screening test, we used a false positive proportion of 42.1% for PSA screening (assuming a cutoff of 4 ng/ml is used to determine patients who receive biopsy) [20]. For a 10-percentage point reduction in the false positive proportion, we would expect savings that equate to 14.7% of the overall cost of biopsies and 3.4% of overall screening, diagnosis, treatment and associated complication costs of the population under study, which would be realized by preventing unnecessary diagnostic biopsies and subsequent treatment and complications. These estimates do not include the potential costs of the new screening test and assume the number of true men identified with prostate cancer remain unchanged, irrespective of the screening tool that is used. Conversely, increasing the true positive rate of the screening test would lead to additional costs to the health system. We estimated that the true positive rate for PSA screening is 28% (e.g., 28% of men with prostate cancer are identified by a PSA cutoff of 4 ng/ml). For a 10-percentage point increase in the PSA screen true positive rate for the study sample, we can expect the cost increase to equate to 35.4% of the overall cost of treatment and 23.6% of the overall cost of screening and treatment compared to the current scenario.

## Discussion

Screening, diagnosis, treatment and associated complication costs from the payer perspective among men 70 years old or older—the group least likely to benefit from the practice [4]—amounted to nearly two thirds of the overall cost. Notably, men in this age group make up less than one third of the patient population in the multispecialty group. Correspondingly, a decrease in screening rate by 5 percentage points in men 70 years and older would result in the largest percent decrease in cost when compared to other age groups. Given that a significant proportion of men in this age group who are screened and are diagnosed with low risk prostate cancer will receive aggressive therapy with questionable benefit, there may be an opportunity to be more selective in patients that receive screening and aggressive treatment [4, 21].

In light of revised national guidelines from the United States Preventive Services Task Force and the American Urological Association combined with the move towards risk-based payment strategies nationally [22–24], health systems and payers are incentivized to align practice with evidence based guidelines. Inputs from our model—including the age distribution of the population, rates of screening, and unit costs—may be easily adapted to be employed by other health care systems.

The increased overall cost to screen and treat men aged 70 years and older are driven by higher incidence of cancer in this population among men who are screened [12]. Ma et al. also found that screening and biopsy for older age groups, particularly the 75-99-year-old age group, in the Medicare population resulted in higher costs, but were not able to assess the cost implications of treatment across age groups [25]. Additionally, the complication rates for biopsy and prostate cancer treatments are substantial, and although the rates of complication are not well characterized by age group, it is reasonable to expect that they may be higher in older populations given higher rates of complicating comorbidities [26, 27], which could be an additional cost driver for this age group.

Ninety percent of costs were attributable to post-screening diagnosis and treatment. With new tests on the horizon that may reduce false positive screens and increase true positive screens specifically for lethal prostate cancer, we explored their theoretical post screening financial impact. Reducing the false positive proportion of the PSA screen would result in modest savings on clinically unnecessary biopsies, but perhaps more importantly, allow patients to avoid the anxiety from falsely positive results and risk of undergoing procedures. Increasing the true positive proportion of screening tests may increase costs as more patients would move into the treatment pathway for the prostate cancer diagnosis. A number of nomograms, molecular biomarkers, and genetic markers currently under study may prevent the need for clinically unnecessary biopsies and treatments as well as better identify which patients would be most likely to benefit from active treatment and which patients would benefit from active surveillance [28–31]. The development and implementation of screening tests that are able to identify potentially lethal prostate cancer will be important in allowing us to more optimally allocate resources in constrained healthcare systems.

This study was designed to determine the costs associated with PSA-based prostate cancer screening in a primary care practice group associated with a large health system in the United States. However, the model we developed is flexible and can use inputs (age distribution of the screen-eligible population, the prevalence of screening, and screening, diagnosis, treatment and associated complications costs) tailored to other health care systems (see online Additional file 3). So while the costs of changing PSA screening practices are specific to our population, our approach and model may be more broadly applied when making decisions regarding the appropriate use of resources. With rising healthcare costs, this tool may provide the potential to use cost data as one piece of evaluating the value of PSA screening and downstream treatments as part of healthcare system clinical practice guidelines. In line with this trend, national clinical organizations such as the American Society of Clinical Oncologists and the American Heart Association have begun to use cost data as one part of evaluating the value of treatments [32, 33].

This study has several possible limitations that warrant discussion. First, our costs reflect reimbursement costs, and while they are an accurate reflection of costs from the payer perspective, they do not take into account other direct and indirect costs that influence total healthcare costs. These other costs include patient out-of-pocket costs, time off from work, and travel costs. Nonetheless, focusing on the payer perspective is important because it may impact how they consider different financial opportunities for interventions. Additionally, we recognize that there are many aspects to the costs associated with evaluating patients with an elevated PSA and treating patients with diagnosed cancer that are joint and may include fixed facility fees, salaried staff costs, and the like. We were unable to accurately assess these joint costs and have not included them in our estimates. In future study, we aim to include both a direct assessment of our own systems costs with screening, diagnosis, and treatment and also evaluation of joint costs. Second, we included costs associated with active treatment for prostate cancer, specifically radiation therapy and radical prostatectomy; in our main scenario, we did not include costs of active surveillance, cryotherapy, or hormone therapy in this analysis due to insufficient claims data to obtain precise cost estimates. With nearly three quarters of patients receiving radiation and/or radical prostatectomy, it is unlikely that excluding these treatments would significantly impact cost projections. Third, the cost of complications was limited to those that occurred shortly after treatment and did not include costs of follow-up visits outside this time frame and intermediate and long-term complications (e.g., erectile dysfunction and urinary incontinence). Given that surgical therapies have higher upfront complications and radiotherapy higher delayed complications, our 90- and 30-day complication windows for surgery and radiotherapy respectively likely under reports overall costs for radiotherapy. Fourth, the 2013-2014 prevalence of PSA screening in screen-eligible men seeking primary care at the physician group were lower than national averages and the rate of PSA screening has changed in recent years following new guidelines [8, 9]. Our model is adaptable to other health care settings and inputs can be adjusted based on institution specific data [34]. In addition, we do not have data on whether men in the sample had been previously screened for prostate cancer, which may impact their likelihood of having a positive/negative test and interval of next screen. Expanding our timeline of analysis may have accounted for these variations in screening but we did not have available data to do so. We also lack data to accurately assess transition probabilities of our sample by racial group and co-morbidity but in future studies hope to include sensitivity analysis across racial groups and different co-morbidity groups [35]. Fifth, in the analysis in which we varied the accuracy of theoretical new prostate cancer screening tests, we did not know the number of patients with true prostate cancer and thus used literature estimates to determine the number of patients undergoing a PSA screen (positive or negative) with true prostate cancer. Though we changed the sensitivity and specificity of theoretical tests, we did not test scenarios in which new tests preferentially identified potentially lethal prostate cancer which may improve the value of screening and subsequent treatment. Lastly, it should be noted that the decision to screen and treat a man for prostate cancer, particularly a man aged 70 years or older, is an individual patient- and provider-level decision and that the assertions from our study cannot be blindly applied without thorough decision-making conversations regarding the pros and cons of PSA screening.

## Conclusions

In a healthcare system with constrained financial resources and national pressure to decrease the rate of PSA screening, there is an opportunity for cost savings by more appropriately identifying patients that will most benefit. To move towards this opportunity, we highlight the importance that targeting men of different age ranges can have on overall costs, in particular by focusing on the downstream costs of potential diagnosis, complications and procedures that may be disproportionately associated with screening older patients.

## Additional files

Additional file 1: Cost model used for our calculations and a generalizable model for other healthcare systems. (XLSX 16 kb)

Additional file 2: Appendix - ICD codes used in cost model as described in methods. (PDF 464 kb)

Additional file 3: Appendix – Description of source data from literature sources used in Node Diagram as part of cost model. (PDF 14 kb)

## Abbreviations

AUA: American Urological Association
CPT: Current Procedural Terminology
ICD: Internal Classification of Diseases
PSA: Prostate specific antigen
USPTF: United States Preventative Task Force
