# Supplementary material for: Cost implications of PSA screening differ by age
Source: BMC Urol. 2018 May 9;18:38. doi: 10.1186/s12894-018-0344-5 (PMC5944051; doi:10.1186/s12894-018-0344-5)
Supplement: Supplementary file 2 — Appendix - ICD codes used in cost model as described in methods. (PDF 464 kb) [file 12894_2018_344_MOESM2_ESM.pdf]

Appendix

Table 1 PSA

PSA test cost may include the following procedures (CPT code)	
84152	ASSAY OF PROSTATE SPECIFIC ANTIGEN COMPLEXED
84154	ASSAY OF PROSTATE SPECIFIC ANTIGEN FREE
84153	ASSAY OF PROSTATE SPECIFIC ANTIGEN TOTAL
36415	COLLECTION VENOUS BLOOD VENIPUNCTURE

Table 2 Biopsy

Biopsy costs may include the following procedures (CPT code)	
55700	PROSTATE NEEDLE BIOPSY ANY APPROACH
76942	US GUIDANCE NEEDLE PLACEMENT RS&I
88305	LEVEL IV SURG PATHOLOGY GROSS&MICROSCOPIC EXAM
76872	ULTRASOUND TRANSRECTAL

Table 3 Biopsy Complication

Criteria 1: Biopsy Complication (Diagnosis Code)		Criteria 2: Hospitalization and ED CPT	
599.7	HEMATURIA	99222	INITIAL HOSPITAL CARE/DAY 50 MINUTES
599.71	GROSS HEMATURIA	99223	INITIAL HOSPITAL CARE/DAY 70 MINUTES
599.72	MICROSCOPIC HEMATURIA	99231	SBSQ HOSPITAL CARE/DAY 15 MINUTES
608.82	HEMATOSPERMIA	99233	SBSQ HOSPITAL CARE/DAY 35 MINUTES
998.59	OTHER POSTOPERATIVE INFECTION NEC	99236	OBSERVATION/INPATIENT HOSPITAL CARE 55 MINUTES
719.41	JOINT PAIN-SHOULD	99238	HOSPITAL DISCHARGE DAY MANAGEMENT 30 MIN/<
719.44	PAIN IN JOINT, HAND	99239	HOSPITAL DISCHARGE DAY MANAGEMENT > 30 MIN
719.45	PAIN IN JOINT PELVIC REGION&THIGH	99281	EMERGENCY DEPARTMENT VISIT LIMITED/MINOR PROB
719.46	JOINT PAIN-LOW LE	99282	EMERGENCY DEPARTMENT VISIT LOW/MODER SEVERITY
719.47	JOINT PAIN-ANKLE	99283	EMERGENCY DEPARTMENT VISIT MODERATE SEVERITY
719.49	PAIN IN JOINT, MULTIPLE SITES	99284	EMERGENCY DEPARTMENT VISIT HIGH/URGENT SEVERITY
724.1	PAIN IN THORACIC SPINE	99285	EMERGENCY DEPT VISIT HIGH SEVERITY&THREAT FUNCJ
729.5	PAIN IN LIMB		
786.5	CHEST PAIN NOS		
786.59	OTHER CHEST PAIN		
789	ABDOMINAL PAIN-SITE NOS		
789.03	ABDOMINAL PAIN RIGHT LOWER QUADRANT		
599	URINARY TRACT INF NOS		

599.6	URINARY OBSTRUCTION NOS	99291	CRITICAL CARE ILL/INJURED PATIENT INIT 30-74 MIN
997.5	URINARY COMP NEC		
996.64	INFECT D/T URETHRAL CATH		
606.9	MALE INFERTILITY NOS		

Table 4 Radical Prostatectomy

Patients with the following CPT codes were identified as radical prostatectomy patients (CPT code)	
PROSTATECTOMY PERINEAL RADICAL	55810
PROSTATECTOMY PERINEAL RADICAL W/LYMPH NODE BX	55812
PROSTATECTOMY PERINEAL RAD W/BI PELVIC LYMPH EXC	55815
PROSTATECTOMY SUPRAPUBIC SUBTOTAL 1/2 STAGES	55821
PROSTATECTOMY RETROPUBIC SUBTOTAL	55831
PROSTATECTOMY RETROPUBIC W/WO NERVE SPARING	55840
PROSTECT RETROPUBIC RAD W/WO NRV SPAR W/LYMPH BX	55842
PROSTECT RETROPUB RAD W/WO NRV SPAR & BI PLV LYM	55845
LAPS PROSTECT RETROPUBIC RAD W/NRV SPARING ROBOT	55866

Table 5 Radical Prostatectomy Complication

Criteria 1: Post Surgery Complication ICD9 Diagnosis			
Other Medical	3530	Brachial plexus lesions	353
Other Medical	3542	Lesion of ulnar nerve	354.2
Cardiac	40201	Malignant hypertensive heart disease with heart failure	402.01
Cardiac	40211	Benign hypertensive heart disease with heart failure	402.11
Cardiac	40291	Unspecified hypertensive heart disease with heart failure	402.91
Cardiac	410	Acute myocardial infarction	410
Cardiac	4100	Acute myocardial infarction of anterolateral wall	410
Cardiac	41000	Acute myocardial infarction of anterolateral wall, episode of care unspecified	410
Cardiac	41001	Acute myocardial infarction of anterolateral wall, initial episode of care	410.01
Cardiac	41002	Acute myocardial infarction of anterolateral wall, subsequent episode of care	410.02
Cardiac	4101	Acute myocardial infarction of other anterior wall	410.1
Cardiac	41010	Acute myocardial infarction of other anterior wall, episode of care unspecified	410.1
Cardiac	41011	Acute myocardial infarction of other anterior wall, initial episode of care	410.11
Cardiac	41012	Acute myocardial infarction of other anterior wall, subsequent episode of care	410.12
Cardiac	4102	Acute myocardial infarction of inferolateral wall	410.2
Cardiac	41020	Acute myocardial infarction of inferolateral wall, episode of care unspecified	410.2
Cardiac	41021	Acute myocardial infarction of inferolateral wall, initial episode of care	410.21
Cardiac	41022	Acute myocardial infarction of inferolateral wall, subsequent episode of care	410.22
Cardiac	4103	Acute myocardial infarction of inferoposterior wall	410.3

Cardiac	41030	Acute myocardial infarction of inferoposterior wall, episode of care unspecified	410.3
Cardiac	41031	Acute myocardial infarction of inferoposterior wall, initial episode of care	410.31
Cardiac	41032	Acute myocardial infarction of inferoposterior wall, subsequent episode of care	410.32
Cardiac	4104	Acute myocardial infarction of other inferior wall	410.4
Cardiac	41040	Acute myocardial infarction of other inferior wall, episode of care unspecified	410.4
Cardiac	41041	Acute myocardial infarction of other inferior wall, initial episode of care	410.41
Cardiac	41042	Acute myocardial infarction of other inferior wall, subsequent episode of care	410.42
Cardiac	4105	Acute myocardial infarction of other lateral wall	410.5
Cardiac	41050	Acute myocardial infarction of other lateral wall, episode of care unspecified	410.5
Cardiac	41051	Acute myocardial infarction of other lateral wall, initial episode of care	410.51
Cardiac	41052	Acute myocardial infarction of other lateral wall, subsequent episode of care	410.52
Cardiac	4106	Acute myocardial infarction, true posterior wall infarction	410.6
Cardiac	41060	True posterior wall infarction, episode of care unspecified	410.6
Cardiac	41061	True posterior wall infarction, initial episode of care	410.61
Cardiac	41062	True posterior wall infarction, subsequent episode of care	410.62
Cardiac	4107	Acute myocardial infarction, subendocardial infarction	410.7
Cardiac	41070	Subendocardial infarction, episode of care unspecified	410.7
Cardiac	41071	Subendocardial infarction, initial episode of care	410.71
Cardiac	41072	Subendocardial infarction, subsequent episode of care	410.72
Cardiac	4108	Acute myocardial infarction of other specified sites	410.8
Cardiac	41080	Acute myocardial infarction of other specified sites, episode of care unspecified	410.8
Cardiac	41081	Acute myocardial infarction of other specified sites, initial episode of care	410.81
Cardiac	41082	Acute myocardial infarction of other specified sites, subsequent episode of care	410.82
Cardiac	4109	Acute myocardial infarction, unspecified site	410.9
Cardiac	41090	Acute myocardial infarction of unspecified site, episode of care unspecified	410.9
Cardiac	41091	Acute myocardial infarction of unspecified site, initial episode of care	410.91
Cardiac	41092	Acute myocardial infarction of unspecified site, subsequent episode of care	410.92
Cardiac	4275	Cardiac arrest	427.5
Cardiac	428	Heart failure	428
Cardiac	4280	Congestive heart failure, unspecified	428
Cardiac	4281	Left heart failure	428.1
Cardiac	42820	Systolic heart failure, unspecified	428.2
Cardiac	42821	Acute systolic heart failure	428.21
Cardiac	42822	Chronic systolic heart failure	428.22
Cardiac	42823	Acute on chronic systolic heart failure	428.23
Cardiac	42830	Diastolic heart failure, unspecified	428.3
Cardiac	42831	Acute diastolic heart failure	428.31
Cardiac	42832	Chronic diastolic heart failure	428.32
Cardiac	42833	Acute on chronic diastolic heart failure	428.33
Cardiac	42840	Combined systolic and diastolic heart failure, unspecified	428.4
Cardiac	42841	Acute combined systolic and diastolic heart failure	428.41
Cardiac	42842	Chronic combined systolic and diastolic heart failure	428.42
Cardiac	42843	Acute on chronic combined systolic and diastolic heart failure	428.43
Cardiac	4289	Heart failure, unspecified	428.9

Vascular	433	Occlusion and stenosis of precerebral arteries	433
Vascular	4330	Occlusion and stenosis of basilar artery	433
Vascular	43300	Occlusion and stenosis of basilar artery without mention of cerebral infarction	433
Vascular	43301	Occlusion and stenosis of basilar artery with cerebral infarction	433.01
Vascular	4331	Occlusion and stenosis of carotid artery	433.1
Vascular	43310	Occlusion and stenosis of carotid artery without mention of cerebral infarction	433.1
Vascular	43311	Occlusion and stenosis of carotid artery with cerebral infarction	433.11
Vascular	4332	Occlusion and stenosis of vertebral artery	433.2
Vascular	43320	Occlusion and stenosis of vertebral artery without mention of cerebral infarction	433.2
Vascular	43321	Occlusion and stenosis of vertebral artery with cerebral infarction	433.21
Vascular	4333	Occlusion and stenosis of multiple and bilateral precerebral arteries	433.3
Vascular	43330	Occlusion and stenosis of multiple and bilateral precerebral arteries without mention of cerebral infarction	433.3
Vascular	43331	Occlusion and stenosis of multiple and bilateral precerebral arteries with cerebral infarction	433.31
Vascular	4338	Occlusion and stenosis of other specified precerebral artery	433.8
Vascular	43380	Occlusion and stenosis of other specified precerebral artery without mention of cerebral infarction	433.8
Vascular	43381	Occlusion and stenosis of other specified precerebral artery with cerebral infarction	433.81
Vascular	4339	Occlusion and stenosis of unspecified precerebral artery	433.9
Vascular	43390	Occlusion and stenosis of unspecified precerebral artery without mention of cerebral infarction	433.9
Vascular	43391	Occlusion and stenosis of unspecified precerebral artery with cerebral infarction	433.91
Vascular	434	Occlusion of cerebral arteries	434
Vascular	4340	Cerebral thrombosis	434
Vascular	43400	Cerebral thrombosis without mention of cerebral infarction	434
Vascular	43401	Cerebral thrombosis with cerebral infarction	434.01
Vascular	4341	Cerebral embolism	434.1
Vascular	43410	Cerebral embolism without mention of cerebral infarction	434.1
Vascular	43411	Cerebral embolism with cerebral infarction	434.11
Vascular	4349	Unspecified cerebral artery occlusion	434.9
Vascular	43490	Cerebral artery occlusion, unspecified without mention of cerebral infarction	434.9
Vascular	43491	Cerebral artery occlusion, unspecified with cerebral infarction	434.91
Vascular	436	Acute, but ill-defined, cerebrovascular disease	436
Vascular	437	Other and ill-defined cerebrovascular disease	437
Vascular	4370	Cerebral atherosclerosis	437
Vascular	4371	Other generalized ischemic cerebrovascular disease	437.1
Vascular	4372	Hypertensive encephalopathy	437.2
Vascular	4373	Cerebral aneurysm, nonruptured	437.3
Vascular	4374	Cerebral arteritis	437.4
Vascular	4375	Moyamoya disease	437.5
Vascular	4376	Nonpyogenic thrombosis of intracranial venous sinus	437.6
Vascular	4377	Transient global amnesia	437.7
Vascular	4378	Other ill-defined cerebrovascular disease	437.8
Vascular	4379	Unspecified cerebrovascular disease	437.9

Vascular	44422	Arterial embolism and thrombosis of lower extremity	444.22
Vascular	44481	Embolism and thrombosis of iliac artery	444.81
Vascular	4511	Phlebitis and thrombophlebitis of deep veins of lower extremities	451.1
Vascular	45111	Phlebitis and thrombophlebitis of femoral vein (deep) (superficial)	451.11
Vascular	45119	Phlebitis and thrombophlebitis of deep veins of lower extremities, other	451.19
Vascular	4512	Phlebitis and thrombophlebitis of lower extremities, unspecified	451.2
Vascular	45181	Phlebitis and thrombophlebitis of iliac vein	451.81
Vascular	4519	Phlebitis and thrombophlebitis of unspecified site	451.9
Vascular	4538	Embolism and thrombosis of other specified veins	453.8
Vascular	4539	Other venous embolism and thrombosis of unspecified site	453.9
Other Medical	4578	Other noninfectious disorders of lymphatic channels	457.8
Respiratory	466	Acute bronchitis and bronchiolitis	466
Respiratory	4660	Acute bronchitis	466
Respiratory	4661	Acute bronchiolitis	466.1
Respiratory	46611	Acute bronchiolitis due to respiratory syncytial virus (RSV)	466.11
Respiratory	46619	Acute bronchiolitis due to other infectious organisms	466.19
Respiratory	480	Viral pneumonia	480
Respiratory	4800	Pneumonia due to adenovirus	480
Respiratory	4801	Pneumonia due to respiratory syncytial virus	480.1
Respiratory	4802	Pneumonia due to parainfluenza virus	480.2
Respiratory	4803	Pneumonia due to SARS-associated coronavirus	480.3
Respiratory	4808	Pneumonia due to other virus not elsewhere classified	480.8
Respiratory	4809	Viral pneumonia, unspecified	480.9
Respiratory	481	Pneumococcal pneumonia [Streptococcus pneumoniae pneumonia]	481
Respiratory	482	Other bacterial pneumonia	482
Respiratory	4820	Pneumonia due to Klebsiella pneumoniae	482
Respiratory	4821	Pneumonia due to Pseudomonas	482.1
Respiratory	4822	Pneumonia due to Hemophilus influenzae [H. influenzae]	482.2
Respiratory	4823	Pneumonia due to Streptococcus	482.3
Respiratory	48230	Pneumonia due to Streptococcus, unspecified	482.3
Respiratory	48231	Pneumonia due to Streptococcus, group A	482.31
Respiratory	48232	Pneumonia due to Streptococcus, group B	482.32
Respiratory	48239	Pneumonia due to other Streptococcus	482.39
Respiratory	4824	Pneumonia due to Staphylococcus	482.4
Respiratory	48240	Pneumonia due to Staphylococcus, unspecified	482.4
Respiratory	48241	Methicillin susceptible pneumonia due to Staphylococcus aureus	482.41
Respiratory	48242	Methicillin resistant pneumonia due to Staphylococcus aureus	482.42
Respiratory	48249	Other Staphylococcus pneumonia	482.49
Respiratory	4828	Pneumonia due to other specified bacteria	482.8
Respiratory	48281	Pneumonia due to anaerobes	482.81
Respiratory	48282	Pneumonia due to escherichia coli [E. coli]	482.82
Respiratory	48283	Pneumonia due to other gram-negative bacteria	482.83
Respiratory	48284	Pneumonia due to Legionnaires' disease	482.84
Respiratory	48289	Pneumonia due to other specified bacteria	482.89
Respiratory	4829	Bacterial pneumonia, unspecified	482.9
Respiratory	483	Pneumonia due to other specified organism	483
Respiratory	4830	Pneumonia due to mycoplasma pneumoniae	483

Respiratory	4831	Pneumonia due to chlamydia	483.1
Respiratory	4838	Pneumonia due to other specified organism	483.8
Respiratory	485	Bronchopneumonia, organism unspecified	485
Respiratory	486	Pneumonia, organism unspecified	486
Respiratory	514	Pulmonary congestion and hypostasis	514
Respiratory	5180	Pulmonary collapse	518
Respiratory	5184	Acute edema of lung, unspecified	518.4
Respiratory	5185	Pulmonary insufficiency following trauma and surgery	518.5
Respiratory	51881	Acute respiratory failure	518.81
Respiratory	51882	Other pulmonary insufficiency, not elsewhere classified	518.82
Other Medical	531	Gastric ulcer	531
Other Medical	5310	Acute gastric ulcer with hemorrhage	531
Other Medical	53100	Acute gastric ulcer with hemorrhage, without mention of obstruction	531
Other Medical	53101	Acute gastric ulcer with hemorrhage, with obstruction	531.01
Other Medical	5311	Acute gastric ulcer with perforation	531.1
Other Medical	53110	Acute gastric ulcer with perforation, without mention of obstruction	531.1
Other Medical	53111	Acute gastric ulcer with perforation, with obstruction	531.11
Other Medical	5312	Acute gastric ulcer with hemorrhage and perforation	531.2
Other Medical	53120	Acute gastric ulcer with hemorrhage and perforation, without mention of obstruction	531.2
Other Medical	53121	Acute gastric ulcer with hemorrhage and perforation, with obstruction	531.21
Other Medical	5313	Acute gastric ulcer without mention of hemorrhage or perforation	531.3
Other Medical	53130	Acute gastric ulcer without mention of hemorrhage or perforation, without mention of obstruction	531.3
Other Medical	53131	Acute gastric ulcer without mention of hemorrhage or perforation, with obstruction	531.31
Other Medical	5314	Chronic or unspecified gastric ulcer with hemorrhage	531.4
Other Medical	53140	Chronic or unspecified gastric ulcer with hemorrhage, without mention of obstruction	531.4
Other Medical	53141	Chronic or unspecified gastric ulcer with hemorrhage, with obstruction	531.41
Other Medical	5315	Chronic or unspecified gastric ulcer with perforation	531.5
Other Medical	53150	Chronic or unspecified gastric ulcer with perforation, without mention of obstruction	531.5
Other Medical	53151	Chronic or unspecified gastric ulcer with perforation, with obstruction	531.51
Other Medical	5316	Chronic or unspecified gastric ulcer with hemorrhage and perforation	531.6
Other Medical	53160	Chronic or unspecified gastric ulcer with hemorrhage and perforation, without mention of obstruction	531.6

Other Medical	53161	Chronic or unspecified gastric ulcer with hemorrhage and perforation, with obstruction	531.61
Other Medical	5317	Chronic gastric ulcer without mention of hemorrhage or perforation	531.7
Other Medical	53170	Chronic gastric ulcer without mention of hemorrhage or perforation, without mention of obstruction	531.7
Other Medical	53171	Chronic gastric ulcer without mention of hemorrhage or perforation, with obstruction	531.71
Other Medical	5319	Gastric ulcer, unspecified as acute or chronic, without mention of hemorrhage or perforation	531.9
Other Medical	53190	Gastric ulcer, unspecified as acute or chronic, without mention of hemorrhage or perforation, without mention of obstruction	531.9
Other Medical	53191	Gastric ulcer, unspecified as acute or chronic, without mention of hemorrhage or perforation, with obstruction	531.91
Other Medical	532	Duodenal ulcer	532
Other Medical	5320	Acute duodenal ulcer with hemorrhage	532
Other Medical	53200	Acute duodenal ulcer with hemorrhage, without mention of obstruction	532
Other Medical	53201	Acute duodenal ulcer with hemorrhage, with obstruction	532.01
Other Medical	5321	Acute duodenal ulcer with perforation	532.1
Other Medical	53210	Acute duodenal ulcer with perforation, without mention of obstruction	532.1
Other Medical	53211	Acute duodenal ulcer with perforation, with obstruction	532.11
Other Medical	5322	Acute duodenal ulcer with hemorrhage and perforation	532.2
Other Medical	53220	Acute duodenal ulcer with hemorrhage and perforation, without mention of obstruction	532.2
Other Medical	53221	Acute duodenal ulcer with hemorrhage and perforation, with obstruction	532.21
Other Medical	5323	Acute duodenal ulcer without mention of hemorrhage or perforation	532.3
Other Medical	53230	Acute duodenal ulcer without mention of hemorrhage or perforation, without mention of obstruction	532.3
Other Medical	53231	Acute duodenal ulcer without mention of hemorrhage or perforation, with obstruction	532.31
Other Medical	5324	Chronic or unspecified duodenal ulcer with hemorrhage	532.4
Other Medical	53240	Chronic or unspecified duodenal ulcer with hemorrhage, without mention of obstruction	532.4
Other Medical	53241	Chronic or unspecified duodenal ulcer with hemorrhage, with obstruction	532.41
Other Medical	5325	Chronic or unspecified duodenal ulcer with perforation	532.5
Other Medical	53250	Chronic or unspecified duodenal ulcer with perforation, without mention of obstruction	532.5
Other Medical	53251	Chronic or unspecified duodenal ulcer with perforation, with obstruction	532.51

Other Medical	5326	Chronic or unspecified duodenal ulcer with hemorrhage and perforation	532.6
Other Medical	53260	Chronic or unspecified duodenal ulcer with hemorrhage and perforation, without mention of obstruction	532.6
Other Medical	53261	Chronic or unspecified duodenal ulcer with hemorrhage and perforation, with obstruction	532.61
Other Medical	5327	Chronic duodenal ulcer without mention of hemorrhage or perforation	532.7
Other Medical	53270	Chronic duodenal ulcer without mention of hemorrhage or perforation, without mention of obstruction	532.7
Other Medical	53271	Chronic duodenal ulcer without mention of hemorrhage or perforation, with obstruction	532.71
Other Medical	5329	Duodenal ulcer, unspecified as acute or chronic, without mention of hemorrhage or perforation	532.9
Other Medical	53290	Duodenal ulcer, unspecified as acute or chronic, without hemorrhage or perforation, without mention of obstruction	532.9
Other Medical	53291	Duodenal ulcer, unspecified as acute or chronic, without mention of hemorrhage or perforation, with obstruction	532.91
Other Medical	533	Peptic ulcer, site unspecified	533
Other Medical	5330	Acute peptic ulcer, unspecified site, with hemorrhage	533
Other Medical	53300	Acute peptic ulcer of unspecified site with hemorrhage, without mention of obstruction	533
Other Medical	53301	Acute peptic ulcer of unspecified site with hemorrhage, with obstruction	533.01
Other Medical	5331	Acute peptic ulcer, unspecified site, with perforation	533.1
Other Medical	53310	Acute peptic ulcer of unspecified site with perforation, without mention of obstruction	533.1
Other Medical	53311	Acute peptic ulcer of unspecified site with perforation, with obstruction	533.11
Other Medical	5332	Acute peptic ulcer, unspecified site, with hemorrhage and perforation	533.2
Other Medical	53320	Acute peptic ulcer of unspecified site with hemorrhage and perforation, without mention of obstruction	533.2
Other Medical	53321	Acute peptic ulcer of unspecified site with hemorrhage and perforation, with obstruction	533.21
Other Medical	5333	Acute peptic ulcer, unspecified site, without mention of hemorrhage and perforation	533.3
Other Medical	53330	Acute peptic ulcer of unspecified site without mention of hemorrhage and perforation, without mention of obstruction	533.3
Other Medical	53331	Acute peptic ulcer of unspecified site without mention of hemorrhage and perforation, with obstruction	533.31
Other Medical	5334	Chronic or unspecified peptic ulcer, unspecified site, with hemorrhage	533.4
Other Medical	53340	Chronic or unspecified peptic ulcer of unspecified site with hemorrhage, without mention of obstruction	533.4
Other Medical	53341	Chronic or unspecified peptic ulcer of unspecified site with hemorrhage, with obstruction	533.41
Other Medical	5335	Chronic or unspecified peptic ulcer, unspecified site, with perforation	533.5

Other Medical	53350	Chronic or unspecified peptic ulcer of unspecified site with perforation, without mention of obstruction	533.5
Other Medical	53351	Chronic or unspecified peptic ulcer of unspecified site with perforation, with obstruction	533.51
Other Medical	5336	Chronic or unspecified peptic ulcer, unspecified site, with hemorrhage and perforation	533.6
Other Medical	53360	Chronic or unspecified peptic ulcer of unspecified site with hemorrhage and perforation, without mention of obstruction	533.6
Other Medical	53361	Chronic or unspecified peptic ulcer of unspecified site with hemorrhage and perforation, with obstruction	533.61
Other Medical	5337	Chronic peptic ulcer, unspecified site, without mention of hemorrhage or perforation	533.7
Other Medical	53370	Chronic peptic ulcer of unspecified site without mention of hemorrhage or perforation, without mention of obstruction	533.7
Other Medical	53371	Chronic peptic ulcer of unspecified site without mention of hemorrhage or perforation, with obstruction	533.71
Other Medical	5339	Peptic ulcer, unspecified site, unspecified as acute or chronic, without mention of hemorrhage or p	533.9
Other Medical	53390	Peptic ulcer of unspecified site, unspecified as acute or chronic, without mention of hemorrhage or perforation, without mention of obstruction	533.9
Other Medical	53391	Peptic ulcer of unspecified site, unspecified as acute or chronic, without mention of hemorrhage or perforation, with obstruction	533.91
Other Medical	5601	Paralytic ileus	560.1
Other Medical	5608	Other specified intestinal obstruction	560.8
Other Medical	56081	Intestinal or peritoneal adhesions with obstruction (postoperative) (postinfection)	560.81
Other Medical	56089	Other specified intestinal obstruction	560.89
Other Medical	5609	Unspecified intestinal obstruction	560.9
Other Surgical	5651	Anal fistula	565.1
Wound	567	Peritonitis and retroperitoneal infections	567
Wound	5670	Peritonitis in infectious diseases classified elsewhere	567
Wound	5671	Pneumococcal peritonitis	567.1
Wound	5672	Other suppurative peritonitis	567.2
Wound	56721	Peritonitis (acute) generalized	567.21
Wound	56722	Peritoneal abscess	567.22
Wound	56723	Spontaneous bacterial peritonitis	567.23
Wound	56729	Other suppurative peritonitis	567.29
Wound	56731	Psoas muscle abscess	567.31
Wound	56738	Other retroperitoneal abscess	567.38
Wound	56739	Other retroperitoneal infections	567.39
Wound	5678	Other specified peritonitis	567.8
Wound	56781	Choleperitonitis	567.81
Wound	56782	Sclerosing mesenteritis	567.82
Wound	56789	Other specified peritonitis	567.89
Wound	5679	Unspecified peritonitis	567.9

Other Surgical	5693	Hemorrhage of rectum and anus	569.3
Other Surgical	5694	Other specified disorders of rectum and anus	569.4
Other Surgical	56941	Ulcer of anus and rectum	569.41
Other Surgical	56942	Anal or rectal pain	569.42
Other Surgical	56943	Anal sphincter tear (healed) (old)	569.43
Other Surgical	56944	Dysplasia of anus	569.44
Other Surgical	56949	Other specified disorders of rectum and anus	569.49
Other Surgical	56983	Perforation of intestine	569.83
Other Medical	5738	Other specified disorders of liver	573.8
Other Medical	584	Acute renal failure	584
Other Medical	5845	Acute kidney failure with lesion of tubular necrosis	584.5
Other Medical	5846	Acute kidney failure with lesion of renal cortical necrosis	584.6
Other Medical	5847	Acute kidney failure with lesion of renal medullary [papillary] necrosis	584.7
Other Medical	5848	Acute kidney failure with other specified pathological lesion in kidney	584.8
Other Medical	5849	Acute kidney failure, unspecified	584.9
Other Medical	586	Renal failure, unspecified	586
Genitourinary	5901	Acute pyelonephritis	590.1
Genitourinary	59010	Acute pyelonephritis without lesion of renal medullary necrosis	590.1
Genitourinary	59011	Acute pyelonephritis with lesion of renal medullary necrosis	590.11
Genitourinary	5902	Renal and perinephric abscess	590.2
Genitourinary	5908	Other pyelonephritis or pyonephrosis, not specified as acute or chronic	590.8
Genitourinary	59080	Pyelonephritis, unspecified	590.8
Genitourinary	59081	Pyelitis or pyelonephritis in diseases classified elsewhere	590.81
Genitourinary	5909	Infection of kidney, unspecified	590.9
Genitourinary	591	Hydronephrosis	591
Genitourinary	5933	Stricture or kinking of ureter	593.3
Genitourinary	5934	Other ureteric obstruction	593.4
Other Medical	5934	Other ureteric obstruction	593.4
Genitourinary	59381	Vascular disorders of kidney	593.81
Genitourinary	59382	Ureteral fistula	593.82
Genitourinary	59589	Other specified types of cystitis	595.89
	596	Bladder Neck Obstruction	596
Genitourinary	5961	Intestinovesical fistula	596.1
Genitourinary	5962	Vesical fistula, not elsewhere classified	596.2

Genitourinary	5966	Rupture of bladder, nontraumatic	596.6
Genitourinary	5991	Urethral fistula	599.1
Other Surgical	6040	Orchitis, epididymitis, and epididymo-orchitis, with abscess	604
Other Medical	7234	Brachial neuritis or radiculitis NOS	723.4
Other Medical	7824	Jaundice, unspecified, not of newborn	782.4
Other Medical	7855	Shock without mention of trauma	785.5
Other Medical	78550	Shock, unspecified	785.5
Other Medical	78551	Cardiogenic shock	785.51
Other Medical	78552	Septic shock	785.52
Other Medical	78559	Other shock without mention of trauma	785.59
Respiratory	7991	Respiratory arrest	799.1
Other Surgical	90250	Injury to iliac vessel(s), unspecified	902.5
Other Surgical	90251	Injury to hypogastric artery	902.51
Other Surgical	90252	Injury to hypogastric vein	902.52
Other Medical	9551	Injury to median nerve	955.1
Other Medical	9553	Injury to radial nerve	955.3
Other Medical	9557	Injury to other specified nerve(s) of shoulder girdle and upper limb	955.7
Other Medical	9558	Injury to multiple nerves of shoulder girdle and upper limb	955.8
Other Medical	9559	Injury to unspecified nerve of shoulder girdle and upper limb	955.9
Other Surgical	9560	Injury to sciatic nerve	956
Other Surgical	9561	Injury to femoral nerve	956.1
Other Surgical	9564	Injury to cutaneous sensory nerve, lower limb	956.4
Other Surgical	9565	Injury to other specified nerve(s) of pelvic girdle and lower limb	956.5
Other Surgical	9568	Injury to multiple nerves of pelvic girdle and lower limb	956.8
Other Surgical	9569	Injury to unspecified nerve of pelvic girdle and lower limb	956.9
	9583	Posttraum wnd infec NEC	958.3
Other Medical	9954	Shock due to anesthesia, not elsewhere classified	995.4
	99591	Sepsis	995.91
	99631	Malfunc urethral cath	996.31

	99639	Malfunc gu dev/graft NEC	996.39
	99659	Malfunc oth device/graft	996.59
	99664	React-indwell urin cath	996.64
	99676	Comp-genitourin dev/grft	996.76
Cardiac	9971	Cardiac complications, not elsewhere classified	997.1
Vascular	9972	Peripheral vascular complications, not elsewhere classified	997.2
Respiratory	9973	Respiratory complications	997.3
Other Medical	9974	Digestive system complications, not elsewhere classified	997.4
	99749	Oth digestv system comp	997.49
Genitourinary	9975	Urinary complications, not elsewhere classified	997.5
Other Medical	9980	Postoperative shock, not elsewhere classified	998
Other Surgical	9981	Hemorrhage or hematoma or seroma complicating procedure, not elsewhere classified	998.1
Other Surgical	99811	Hemorrhage complicating a procedure	998.11
Other Surgical	99812	Hematoma complicating a procedure	998.12
Other Surgical	99813	Seroma complicating a procedure	998.13
Other Surgical	9982	Accidental puncture or laceration during a procedure, not elsewhere classified	998.2
Wound	9983	Disruption of operation wound	998.3
	99831	Disrup internal op wound	998.31
Other Surgical	9984	Foreign body accidentally left during a procedure	998.4
Wound	9985	Postoperative infection, not elsewhere classified	998.5
Wound	99851	Infected postoperative seroma	998.51
Wound	99859	Other postoperative infection	998.59
Wound	9986	Persistent postoperative fistula	998.6
Other Medical	9987	Acute reaction to foreign substance accidentally left during a procedure	998.7
Other Surgical	99883	Non-healing surgical wound	998.83
	99889	Oth spcf cmplc procd NEC	998.89
Other Surgical	9989	Unspecified complication of procedure, not elsewhere classified	998.9
Vascular	9992	Other vascular complications of medical care, not elsewhere classified	999.2
Other Medical	9994	Anaphylactic shock due to serum	999.4
Other Medical	9995	Other serum reaction, not elsewhere classified	999.5
Other Medical	9996	Abo incompatibility reaction, not elsewhere classified	999.6
Other Medical	9997	Rh incompatibility reaction, not elsewhere classified	999.7
Other Medical	9998	Other transfusion reaction, not elsewhere classified	999.8
Other Surgical	E8700	Accidental cut, puncture, perforation or hemorrhage during surgical operation	E870.0

Other Surgical	E8704	Accidental cut, puncture, perforation or hemorrhage during endoscopic examination	E870.4
Other Surgical	E8707	Accidental cut, puncture, perforation or hemorrhage during administration of enema	E870.7
Other Surgical	E8708	Accidental cut, puncture, perforation or hemorrhage during other specified medical care	E870.8
Other Surgical	E8709	Accidental cut, puncture, perforation or hemorrhage during unspecified medical care	E870.9
Other Surgical	E8710	Foreign object left in body during surgical operation	E871.0
Other Surgical	E8730	Excessive amount of blood or other fluid during transfusion or infusion	E873.0
Other Surgical	E8760	Mismatched blood in transfusion	E876.0

Criteria 2: Post Surgery Complication Requiring Procedure (CPT)	
50040	DRAINAGE OF KIDNEY
50120	EXPLORATION OF KIDNEY
50125	EXPLORE AND DRAIN KIDNEY
50395	CREATE PASSAGE TO KIDNEY
50398	CHANGE KIDNEY TUBE
50605	INSERT URETERAL SUPPORT
52290	CYSTOSCOPY AND TREATMENT
52332	CYSTOSCOPY AND TREATMENT
52334	CREATE PASSAGE TO KIDNEY
50600	EXPLORATION OF URETER
50700	REVISION OF URETER
50715	RELEASE OF URETER
50760	FUSION OF URETERS
50770	SPLICING OF URETERS
50780	REIMPLANT URETER IN BLADDER
50782	REIMPLANT URETER IN BLADDER
50783	REIMPLANT URETER IN BLADDER
50785	REIMPLANT URETER IN BLADDER
50800	IMPLANT URETER IN BOWEL
50810	FUSION OF URETER & BOWEL
50815	URINE SHUNT TO INTESTINE
50820	CONSTRUCT BOWEL BLADDER
50825	CONSTRUCT BOWEL BLADDER
50840	REPLACE URETER BY BOWEL
50900	REPAIR OF URETER
50940	RELEASE OF URETER

26990	DRAINAGE OF PELVIS LESION
45020	DRAINAGE OF RECTAL ABSCESS
49060	DRAIN OPEN RETROP ABSCESS
51080	DRAINAGE OF BLADDER ABSCESS
52000	Cystourethoroscopy

Table 6 Radiation Therapy

Only patients with more than five radiation oncology treatments are included to calculate the average cost of treatment to the insurer.

Criteria for Radiation Oncology Procedures (CPT Code)	
76873	US TRANSRCT PRSTATE VOL BRACHYTX PLNNING SPX
77263	THERAPEUTIC RADIOLOGY TX PLANNING COMPLEX
77470	SPECIAL TREATMENT PROCEDURE
77370	SPEC MEDICAL RADJ PHYSICS CONSLTJ
77336	CONTINUING MEDICAL PHYSICS CONSLTJ PR WK
77290	THER RAD SIMULAJ-AIDED FIELD SETTING COMPLEX
77280	THER RAD SIMULAJ-AIDED FIELD SETTING SIMPLE
77300	BASIC RADIATION DOSIMETRY CALCULATION
C1717	Brachytx, non-strm HDR Ir
77790	SUPERVISION HANDLING LOADING RADIATION SOURCE
77732	Treatment Device
77301	NTSTY MODUL RADTHX PLN DOSE-VOL HISTOS
77334	TX DEVICES DESIGN & CONSTRUCTION COMPLEX
77338	MLC IMRT DESIGN & CONSTRUCTION PER IMRT PLAN
77418	NTSTY MODUL DLVR 1/MLT FLDS/ARCS PR TX SESSION
77014	CT GUIDANCE RADIATION THERAPY FLDS PLACEMENT
77427	RADIATION TREATMENT MANAGEMENT 5 TREATMENTS
77295	THER RAD SIMULAJ-AIDED FLD SETTING 3-DIMENSIONAL
77331	SPEC DOSIM ONLY PRESCRIBED TREATING PHYS
77417	THERAPEUTIC RADIOLOGY PORT FILMS
77326	BRACHYTHERAPY ISODOSE PLAN SIMPLE
77327	BRACHYTHERAPY ISODOSE PLAN INTERMEDIATE
77328	BRACHYTHERAPY ISODOSE PLAN COMPLEX
77785	REMOTE AFTLD RADIONUCLIDE BRACHYTX 1 CHANNEL
77786	REMOTE AFTLD RADIONUCLIDE BRACHYTX 2-12 CHANNEL
77787	REMOTE AFTLD RADIONUCLIDE BRACHYTX > 12 CHANNEL
77403	RADJ DLVR 1 AREA 1/PRLL OPSD PORTS SMPL 6-10MEV
77404	RADJ DLVR 1 AREA 1/PRLL OPSD PORTS SMPL 11-19MEV
77405	DAI MEGAVOLTAGE RX MGMT; INTERME
77406	RADJ DLVR 1 AREA 1/PRLL OPSD PORTS SMPL 20MEV/<
77407	RADJ DLVR 2 AREAS 3/>PORTS 1 MLT BLKS <5MEV
77408	RADJ DLVR 2 AREAS 3/>PORTS 1 MLT BLKS 6-1MEV
77409	RADJ DLVR 2 AREAS 3/>PORTS 1 MLT BLKS 11-19MEV
77410	DAI MEGAVOLTAGE RX MGMT; COMPLEX

77411	RADJ DLVR 2 AREAS 3/> PORTS 1 TX AREA 20 MEV/<
77412	RADJ DLVR 3/> AREAS CUSTOM BLKING <5MEV
77413	RADJ DLVR 3/> AREAS CUSTOM BLKING 6-10MEV
77414	RADJ DLVR 3/> AREAS CUSTOM BLKING 11-19MEV
77415	THER RAD RX PORT FILM INTERPRET
77416	RADJ DLVR 3/> AREAS CUSTOM BLKING 20MEV/<
And: 77421 Or "76950" Or "76370" Or "77522" Or "77332" Or "77778" Or "76965" Or "55875"	

Table 7 Radiation Therapy Complications

1) Extrapolated from the EHP database all diagnosis codes for Prostate Cancer Rad Onc patients
2) Selected diagnosis codes for common Rad Onc complications that usually occur within 30 days of treatment.

Criteria for common diagnosis codes for common radiation oncology complications present in our patient sample	
599.71	GROSS HEMATURIA
788.1	DYSURIA
599.7	HEMATURIA UNSPECIFIED
