# Supplementary material for: Cost implications of PSA screening differ by age
Source: BMC Urol. 2018 May 9;18:38. doi: 10.1186/s12894-018-0344-5 (PMC5944051; doi:10.1186/s12894-018-0344-5)
Supplement: Supplementary file 3 — Appendix – Description of source data from literature sources used in Node Diagram as part of cost model. (PDF 14 kb) [file 12894_2018_344_MOESM3_ESM.pdf]

Appendix:

Source data for node transitions:

Node 1: PSA screening practice data from the Johns Hopkins Community Physicians group from April 1st 2013 to March 31st, 2014.

Node 2: PSA data obtained on a nationally representative sample of American men 40 years of age and older with no history of prostate cancer and no current inflammation or infection of the prostate gland (n = 1308) from the 2001-2002 National Health and Nutrition Examination Survey.[11]

Node 3: 38,350 men that were randomly assigned to the screening arm of the PLCO trial from November 1993 to June 2001. [12]

Node 4: 38,350 men that were randomly assigned to the screening arm of the PLCO trial from November 1993 to June 2001.[12]

Node 5: 13,805 men enrolled in the Cancer of the Prostate Strategic Urologic Research Endeavor, a national disease registry of men with biopsy proven prostate adenocarcinoma.[13]

Node 6: 13,805 men enrolled in the Cancer of the Prostate Strategic Urologic Research Endeavor, a national disease registry of men with biopsy proven prostate adenocarcinoma.
